# Impact of particle flux on the vertical distribution and diversity of size-fractionated prokaryotic communities in two East Antarctic polynyas

**DOI:** 10.3389/fmicb.2023.1078469

**Published:** 2023-02-23

**Authors:** Viena Puigcorbé, Clara Ruiz-González, Pere Masqué, Josep M. Gasol

**Affiliations:** ^1^Department of Marine Biology and Oceanography, Institut de Ciències del Mar (ICM-CSIC), Barcelona, Catalunya, Spain; ^2^Centre for Marine Ecosystems Research, School of Science, Edith Cowan University, Joondalup, WA, Australia; ^3^International Atomic Energy Agency, City of Monaco, Monaco

**Keywords:** prokaryotic communities, marine particles, ^234^Thorium, particle export, polynyas, Antarctica, particle size fractionation, particle-attached and free-living prokaryotes

## Abstract

Antarctic polynyas are highly productive open water areas surrounded by ice where extensive phytoplankton blooms occur, but little is known about how these surface blooms influence carbon fluxes and prokaryotic communities from deeper waters. By sequencing the 16S rRNA gene, we explored the vertical connectivity of the prokaryotic assemblages associated with particles of three different sizes in two polynyas with different surface productivity, and we linked it to the magnitude of the particle export fluxes measured using thorium-234 (^234^Th) as particle tracer. Between the sunlit and the mesopelagic layers (700 m depth), we observed compositional changes in the prokaryotic communities associated with the three size-fractions, which were mostly dominated by *Flavobacteriia*, *Alphaproteobacteria*, and *Gammaproteobacteria*. Interestingly, the vertical differences between bacterial communities attached to the largest particles decreased with increasing ^234^Th export fluxes, indicating a more intense downward transport of surface prokaryotes in the most productive polynya. This was accompanied by a higher proportion of surface prokaryotic taxa detected in deep particle-attached microbial communities in the station with the highest ^234^Th export flux. Our results support recent studies evidencing links between surface productivity and deep prokaryotic communities and provide the first evidence of sinking particles acting as vectors of microbial diversity to depth in Antarctic polynyas, highlighting the direct influence of particle export in shaping the prokaryotic communities of mesopelagic waters.

## 1. Introduction

The Southern Ocean (<35°S) is a key player in the global carbon cycle and the regulation of the Earth’s climate, accounting for ~40% of the anthropogenic CO_2_ oceanic uptake (Gruber et al., 2009 and references therein). Although the Southern Ocean is the largest high-nutrient low-chlorophyll region, highly biologically productive areas are found south of the circumpolar current (del Castillo et al., 2019), particularly on the continental shelf, in coastal zones including polynyas (Sedwick and DiTullio, 1997; Montes-Hugo and Yuan, 2012; Arrigo et al., 2015). Polynyas are ice-free areas created and maintained by katabatic winds and surrounded by consolidated sea ice. In the polynyas, solar radiation reaches the water column and, when it becomes stratified, light together with nutrient inputs from different sources (e.g., Lannuzel et al., 2010; de Jong et al., 2012; Shadwick et al., 2013) triggers significant phytoplankton blooms (Kang et al., 2001; Arrigo et al., 2015; Jena and Pillai, 2020). This enhanced primary productivity can lead to high local carbon export rates mediated by the sinking of organic particles, thus potentially contributing to the ocean uptake of atmospheric CO_2_ (Hoppema and Anderson, 2007; Miller and DiTullio, 2007; Arrigo et al., 2008). However, the timing, extent, and intensity of phytoplankton blooms and primary production can vary significantly even amongst closely located Antarctic polynyas (Arrigo et al., 2015; Moreau et al., 2019), with potential but poorly known implications for the efficiency of the export flux and for the underlying microbial communities that depend on surface-derived carbon inputs.

The structure of surface phytoplankton communities determines the amount, quality, and sinking rates of particles leaving the surface ocean. Besides, physical processes and complex food web interactions, including remineralization processes driven by bacteria, modulate the export and transfer efficiency of the biological carbon pump (Henson et al., 2019; Wiedmann et al., 2020; Nguyen et al., 2022). In turn, sinking particles have been shown to act as microbial diversity vectors between the surface waters and the deep ocean, delivering particle-attached microorganisms that seem able to colonize deeper waters (Mestre et al., 2018), and explaining why meso- and bathypelagic bacterial communities associated with different size-fractions reflect surface gradients of surface phytoplankton productivity (Ruiz-González et al., 2020). In coastal polar ecosystems, pelagic bacteria have been shown to respond quickly to spring and summertime phytoplankton blooms, although the fraction of primary production consumed by heterotrophic bacteria seems highly variable (Ducklow and Yager, 2007; Kirchman et al., 2009; Luria et al., 2016), which could be one of the reasons behind the inverse relationship between primary production and export efficiency often observed in the Southern Ocean (Maiti et al., 2013; le Moigne et al., 2016; Henson et al., 2019). This suggests that particle export fluxes and the interacting prokaryote assemblages are tightly dependent on each other. However, very few studies have coupled measurements of particle export with particle-attached prokaryotic community composition, especially in Antarctic polynyas.

Sinking particle fluxes in the ocean can be directly measured by using sediment traps, which have some advantages (e.g., temporal coverage in the case of moored sediment traps) but have a limited spatial coverage and are time consuming to deploy/recover. Therefore, indirect methods are essential to increase the number of observations across the ocean. One of the most common indirect methods for quantifying the magnitude of sinking particle fluxes in the ocean is the use of parent-daughter radionuclide pairs. Among them, the ^234^Th/^238^U pair is the most extensively used (Ceballos-Romero et al., 2022). ^234^Th is continuously produced by the decay of ^238^U, which has a conservative behavior in oxygenated ocean waters, and is highly particle reactive, so it can be used as a particle tracer. Moreover, the relatively short half-life of ^234^Th (T_1/2_ = 24.1 d) suits the biologically mediated temporal changes in particle production and export. ^238^U, on the other hand, has a very long half-life (T_1/2_ = 4.5 10^9^ y). The difference in half-lives between ^234^Th and ^238^U implies that both radioisotopes should be in secular equilibrium (i.e., ^234^Th/^238^U activity ratio of 1) in the environment. However, in the presence of marine particles, ^234^Th sorbs onto them and can be scavenged when the particles sink, thus breaking the secular equilibrium in the water column (i.e., ^234^Th/^238^U activity ratio <1). The magnitude of the disequilibrium between both radioisotopes allows estimating the magnitude of the particle export flux, which can be converted to fluxes of particulate organic carbon, trace metals, or pollutants (Gustafsson et al., 1997; Black et al., 2019; Puigcorbé et al., 2020; Tesán Onrubia et al., 2020). Thus, combining ^234^Th fluxes with vertical variations in the composition of the communities attached to sinking particles can provide a direct way to link surface productivity, export fluxes, and impacts on microbial communities.

Here, we explore whether differences in surface productivity among Antarctic polynyas impact differentially the particle export fluxes and the microbial communities from the underlying mesopelagic waters. To do so, prokaryotic communities associated with particles of three different sizes were sampled in three stations located in two East Antarctic polynyas differing largely in their surface productivity and dominant phytoplankton groups. We examined the variability in the prokaryotic communities associated with free-living (0.2–0.8 μm), small (0.8–53 μm), and large (>53 μm) particles collected along the water column and compared them to the magnitude of the particle export fluxes estimated using thorium-234 (^234^Th) as particle tracer. We hypothesize that mesopelagic prokaryotic communities will be more similar to those in the surface in the stations with the highest surface productivity and higher particle export fluxes.

## 2. Materials and methods

### 2.1. Sampling and analyses of physicochemical and biological parameters of the study area

Sampling was performed on board of the RSV Aurora Australis (AU1602, AA-V02 2016/17) between 8 December 2016 and 21 January 2017, in one station belonging to the Dalton polynya (D02) and two stations (M36 and M48) located within the Mertz polynya (East Antarctica; 67.2–66.8 ^°^S and 119.5–145.8°E; Supplementary Figure S1A). These stations were chosen due to their contrasting biological, chemical, and physical characteristics, extensively described and discussed by Moreau et al. (2019) and Ratnarajah et al. (2022). For comparison with Ratnarajah et al. (2022), D02 refers to St.2; M36 is EM03 and M48 is MG08. Briefly, both polynyas had similar sea surface temperatures ranging majorly between 0.0 and 1.5°C, but the Dalton polynya showed higher sea surface salinity than the Mertz polynya (34.0–34.3 g/kg vs. 32.5–33.5 g/kg, respectively), suggesting that the later could have experienced more sea ice melting than the Dalton polynya (Moreau et al., 2019). The Dalton polynya presented deeper euphotic depths (95 ± 56 m) and mixed layer depths (25 ± 12 m, excluding two stations where the mixed layer was down to 100 m and 154 m) than the Mertz polynya (40 ± 9 m and 13 ± 1 m, respectively) (Ratnarajah et al., 2022). In general, chlorophyll-a (Chl-a) concentrations in the surface were higher in the Dalton polynya compared to the Mertz polynya (max of 15 μg L^−1^ vs. 8 μg L^−1^), but Mertz presented a subsurface Chl-a maximum of ~10 μg L^−1^ located between 20 and 70 m depth that was consistent along the whole polynya, whereas in the Dalton that layer was more variable (Moreau et al., 2019). Both polynyas also presented global differences in nutrient ratios (i.e., Si:N or N:P) suggesting different nutrient sources (i.e., water masses) and different phytoplanktonic communities, something which was also corroborated by microscope analyses (Moreau et al., 2019). During the time of sampling, the dominant phytoplankton groups differed between both polynyas, with *Phaeocystis antarctica* dominating in the Dalton station and diatoms dominating in the much more productive Mertz stations (Moreau et al., 2019).

The sampling and analyses of physicochemical and environmental parameters were performed as described in Moreau et al. (2019) and Ratnarajah et al. (2022). Temperature and salinity were obtained from the CTD (Rosenberg and Rintoul, 2017) and fluorescence values were obtained with a fluorometer (ECO-AFL/FL 756, Wetlabs, United States) that was installed also on the CTD rosette. Of particular interest for this study is the concentration of Chl-a, particulate organic carbon (POC) and inorganic nutrients. Chl-a concentrations were obtained from ~500 ml of filtered seawater, extracted with acetone and stored at −20°C for 24–48 h in the dark prior to analysis with a Turner Trilogy fluorometer. POC was determined following Knap et al. (1996) and analyzed using a Thermo Finnigan EA 1112 Series Flash Elemental Analyzer. Concentrations of inorganic macronutrients (nitrate, nitrite and silicic acid) were analyzed after the voyage at the CSIRO laboratory (Hobart, Australia) following the methods described in Murphy and Riley (1962), Armstrong et al. (1967), Wood et al. (1967) and Kérouel and Aminot (1997).

### 2.2. Sampling of free-living and particle-attached prokaryotic communities

Seawater samples were collected using 12 L Niskin bottles attached to a CTD rosette. At each station, 6 depths were sampled covering the entire water column (down to ~50 m above the bottom, see Table 1). After collection, the samples (5–11 L) were filtered onboard at 4°C using a Masterflex peristaltic pump. The filtration was done sequentially through a 53 μm pore-size Nitex screen mesh, followed by a 0.8 μm pore-size polycarbonate membrane filter (Millipore) and finally through a 0.2 μm Sterivex filter, thus obtaining three size-fractions from each sample: >53 μm, 0.8–53 μm and 0.2–0.8 μm (for simplification, hereafter 53 μm, 0.8 μm, and 0.2 μm). After filtration, the filters were stored at −80°C for further analyses at the home laboratory.

**Table 1:** Characteristics of the sampled sites—location, date, depth, and filtered volume for DNA samples per each size-fraction.

		Depth (m)	Sample volume (L)		
			53 μm	0.8 μm	0.2 μm
Polynya	Dalton	5	6.5*	6.5	5.0
Station	D02	20	9.1	9.1	5.0
Lat (°S)	66.843	100	7.5*	7.5	7.5
Lon (°E)	119.543	300	8.0*	8.0	3.0
Collection date	31/Dec/16	550	8.0	8.0	2.5
		720	9.0	9.0	2.5
Polynya	Mertz	5	7.8	7.8	7.8
Station	M36	25	9.4	9.4	5.0
Lat (°S)	66.908	100	10.6	10.4	5.0
Lon (°E)	145.498	300	9.8	9.8	5.0
Collection date	10/Jan/17	550	10.2	9.8	5.0
		638	10.7	10.5	5.0
Polynya	Mertz	5	5.9	5.9	5.0
Station	M48	50	9.3	5.0	3.8
Lat (°S)	67.219	100	10.2	10.1	3.0
Lon (°E)	145.881	300	10.6	10.6	5.0
Collection date	11/Jan/17	500	9.4	9.4	5.0
		670	4.9	4.9	4.7

*Stations from where sequencing data are not available due to low DNA recoveries.

### 2.3. Particle export fluxes derived from the ^238^U-^234^Th method

^234^Th analyses are described in Ratnarajah et al. (2022). Briefly, 4 L seawater samples were collected at 12–14 depths along the water column at each station and processed using the manganese oxide co-precipitation technique (Clevenger et al., 2021), while ^238^U activity concentrations were derived from salinity data (Owens et al., 2011). Th-234 samples were counted onboard using a gas flow proportional low-level RISO beta counter (counting statistics <5%) and recounted >6 months later to account for background activities. Chemical recoveries were obtained following Puigcorbé et al. (2017a) and measured by inductively coupled plasma mass spectrometry at the Alfred Wegener Institute.

^234^Th export fluxes (proxies of particle fluxes) were estimated by integrating the ^234^Th deficit in the upper water column relative to ^238^U, using a 1D scavenging model assuming steady state conditions and no significant advection nor diffusion transport. The integration depth used here is the depth where ^234^Th and ^238^U reached secular equilibrium (i.e., ^234^Th/^238^U activity ratio = 1) (see Ratnarajah et al. (2022) for further details).

### 2.4. Characterization of prokaryotic communities

Prokaryotic community structure was determined by high-throughput Illumina sequencing of the 16S rRNA genes. A total of 54 samples were obtained, which were stored at −80°C until analyses were conducted. At the home laboratory, DNA was extracted using the PowerWater DNA Isolation kit following manufacturer’s instructions (MOBIO). The samples were sequenced using Illumina MiSeq 2 × 300 bp flow cells at RTL Genomics (Texas, United States) using primers 515F-Y and 926R (Parada et al., 2015) to amplify the V4-V5 region of the 16S rRNA gene. Three of the 53 μm samples from the Dalton polynya did not have enough DNA material and could not be sequenced (see Table 1). The sequences were processed according to Logares (2017). In brief, primers were removed with Cutadapt (Martin, 2011). The paired-end reads were merged with PEAR (Zhang et al., 2014). Quality filtering, chimera checking and operational taxonomic unit (OTU) clustering (99% similarity) were done with the UPARSE pipeline (Edgar, 2013). Singletons and chimeric OTUs were removed, and the remaining OTUs were taxonomically annotated using the SILVA v123 database. OTUs assigned to chloroplasts were removed, resulting in a total of 8,219 OTUs and 402,440 sequences. To enable comparisons between samples, the OTU table was randomly subsampled to ensure an equal number of sequences per sample (5,000 sequences) using *rrarefy* (Vegan package, R Core Team, 2017), retaining 253,545 sequences clustered into 6,546 OTUs. The raw sequence data have been deposited in the Figshare data repository, doi: 10.6084/m9.figshare.21385215.v1.

### 2.5. Statistical analyses

The spatial differences between prokaryotic communities were visualized using nonmetric multidimensional scaling (NMDS, Vegan *metaMDS* function) based on Bray–Curtis distances. Significant differences in taxonomic composition between depths, stations, or size-fraction were tested using ANOSIM (Vegan *anosim* function). Vertical differences between the prokaryotic communities within each size-fraction were estimated for each individual station as the Bray–Curtis dissimilarity between the surface (5 m) and each of the deeper prokaryotic communities, and the proportion of “surface-derived” OTUs in mesopelagic communities was estimated considering those mesopelagic OTUs that showed presence in surface (<100 m) waters. OTUs unique to a given sampling station (i.e., OTUs present exclusively in one of the three sampled stations) were identified considering all depths together within each station. Statistical analyses and data handling were done in R (R Core Team, 2017).

## 3. Results

### 3.1. Overview of the physicochemical and biological conditions

The two polynyas differed largely in their physicochemical conditions and surface productivity, which have been described in more detail in Moreau et al. (2019) and Ratnarajah et al. (2022) and are summarized in section 2.1. In the three sampled stations, we observed a much larger fluorescence peak (proxy of phytoplankton concentrations) at the two Mertz stations (M36 and M48), compared to the Dalton station (D02), coincident with peaks in ammonia (usually produced in the euphotic zone by heterotrophic bacteria and zooplankton grazing; Smith et al., 2022, and references therein) which were also clearly higher in the Mertz stations than in the Dalton one. Surface concentrations of H_4_SiO_4,_ PO_4_^−3^, and NO_3_ were much lower in the two Mertz stations than in Dalton surface waters, which was suggested to be due to the higher consumption by phytoplankton, particularly diatoms (Moreau et al., 2019; Ratnarajah et al., 2022) (Supplementary Figure S1).

**Figure 1:**
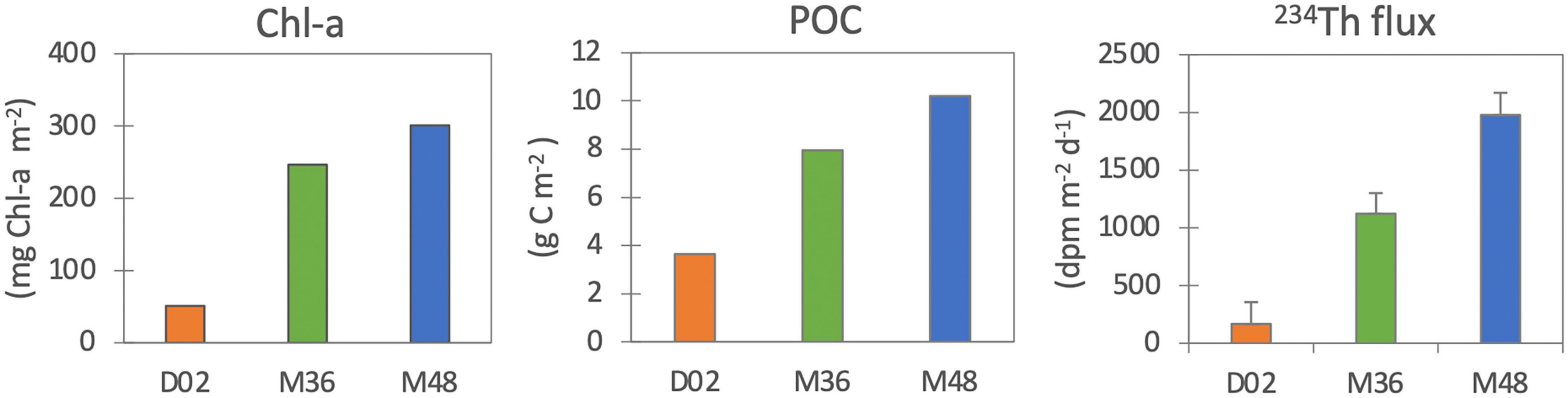
Chlorophyll-a (mg Chl-a m^−2^), particulate organic carbon (mg C m^−2^) stocks in the upper water column (upper 20 m) and ^234^Th export fluxes (dpm m^−2^ d^−1^).

The Chl-a stocks (in the upper 20 m) were much lower in Dalton (51 mg Chl-a m^−2^) than in the M36 and M48 Mertz stations (243 mg Chl-a m^−2^ and 300 mg Chl-a m^−2^, respectively, Figure 1). Accordingly, POC stocks were also lowest at D02 (3.6 mg C m^−2^) compared to M36 (8.0 mg C m^−2^) and M48 (10.2 mg C m^−2^). ^234^Th export fluxes (indicative of sinking particle fluxes), as expected, followed the same trend of Chl-a and POC stocks and ranged from 167 dpm m^−2^ d^−1^ in D02, to 1,122 dpm m^−2^ d^−1^ and 1977 dpm m^−2^ d^−1^ at M36 and M48, respectively (Figure 1).

### 3.2. Vertical variations in taxonomic richness and composition of prokaryotic communities

**Figure 2:**
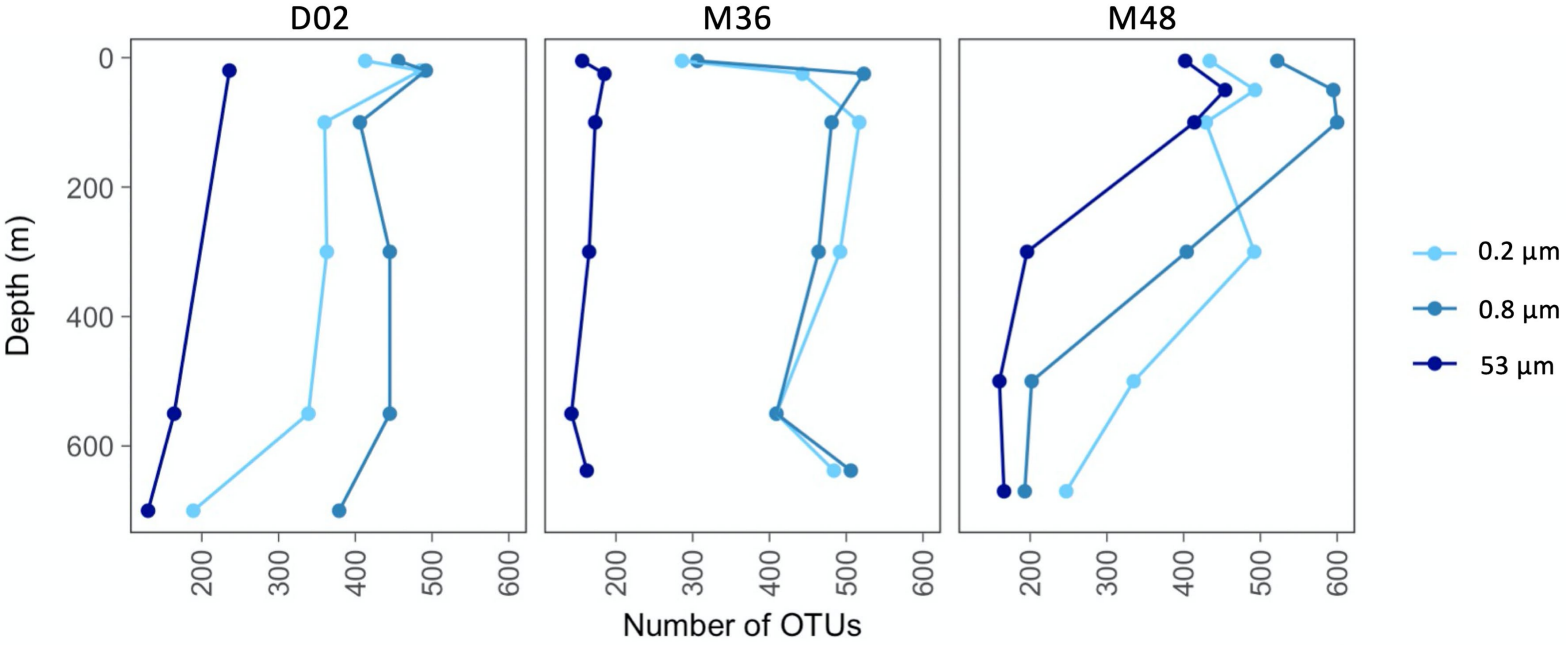
Vertical variations in taxonomic richness (number of OTUs per community) across stations and size-fractions.

The number of observed OTUs ranged between 130 and 600 across the sampled sites and depths, being lower in communities from the largest particles than in the two other size-fractions (Figure 2). In all studied communities, there was an increase in richness from the most superficial sample to the depth of maximum Chl-a, which was most pronounced in the two smallest size-fractions from M36. In stations D02 and M48, the taxonomic richness decreased from subsurface to deeper waters in the three size-fractions, although this reduction in the number of OTUs was more pronounced in station M48. The richness of the communities from station M36 remained relatively constant throughout the water column below 100 m depth, except for an increase in richness at the deepest site in the two smallest size-fractions (Figure 2). It is also worth noticing that at station M48, which was the station with the highest export flux, the richness of communities from the large particles was much more similar to the richness of the smaller size-fractions, whereas at stations D02 and M36, the large fractions showed markedly lower number of OTUs along the entire water column.

**Figure 3:**
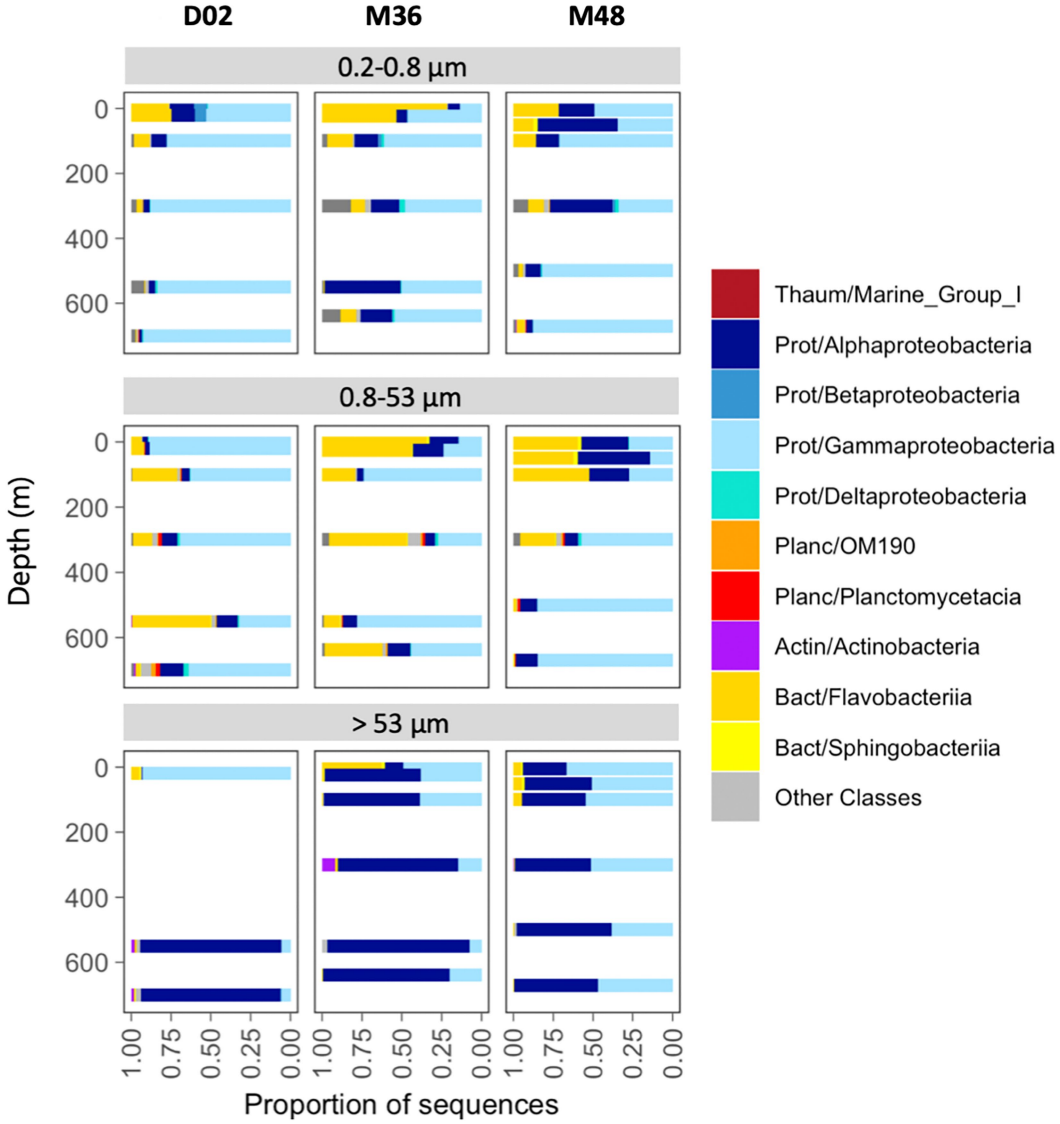
Taxonomic composition across size-fractions and depth in the three stations. The classification was performed at the class level, indicating the phylum: Thaum, thaumarchaeota; Actin, actinobacteria; Bact, bacteroidetes; Planc, planctomycetes; Prot, proteobacteria.

Overall, the communities were dominated by class *Gammaproteobacteria* (53% of total reads), followed by *Alphaproteobacteria* (26%), *Flavobacteriia* (17%), and the Thaumarchaeota Marine Group I (1.5%). Less than 2% of the sequences were classified as Archaea and < 0.05% as Eukaryotes. Communities from the two smallest size-fractions were generally dominated by *Gammaproteobacteria* and/or *Flavobacteriia* across the three stations, whereas *Alphaproteobacteria* accounted for the majority of the sequences in most assemblages associated with the largest particles (Figure 3).

The relative contribution of these groups also changed vertically. In the free-living size-fraction (0.2 μm−0.8 μm), *Flavobacteriia* decreased their abundances from the surface to mesopelagic waters, where communities comprised mostly *Gammaproteobacteria*. *Alphaproteobacteria* decreased with depth in stations D02 and M48 but increased in M36. Particles of intermediate size were dominated mostly by *Gammaproteobacteria* and *Flavobacteriia*, but their vertical patterns differed across stations. Finally, communities associated with the largest particles showed much higher proportions of *Alphaproteobacteria*, which increased their abundances toward mesopelagic waters in the three stations. The largest vertical variations were observed in D02, where communities changed from a dominance of *Gammaproteobacteria* (95% of the sequences) in the surface toward mesopelagic assemblages comprising mostly *Alphaproteobacteria* (~94% of community sequences, Figure 3). Classes like Deltaproteobacteria, OM190, Planctomycetacia, Actinobacteria, and Sphingobacteriia were also detected locally but at much lower abundances (Figure 3).

At the genera level, the communities were more variable across stations and size-fractions (Supplementary Figure S2). Some groups like *Colwellia*, *Pseudoalteromonas*, *Balneatrix* (*Gammaproteobacteria*), and *Polaribacter* (*Flavobacteriia*) were relatively common and present in many of the studied communities, although at varying proportions with depth and size-fraction. Other groups, such as the *Alphaproteobacteria*, *Brevundimonas* or *Sphingorhabdus*, were present only in specific samples, such as in the mesopelagic large particles of D02, where together they accounted for most of the community sequences. Conversely, *Pseudophaeobacter* (*Alphaproteobacteria*) and *Alcanivorax* (*Gammaproteobacteria*) were mostly found associated with the large particles in the two Mertz stations along most of the sampled depths (Supplementary Figure S2).

**Figure 4:**
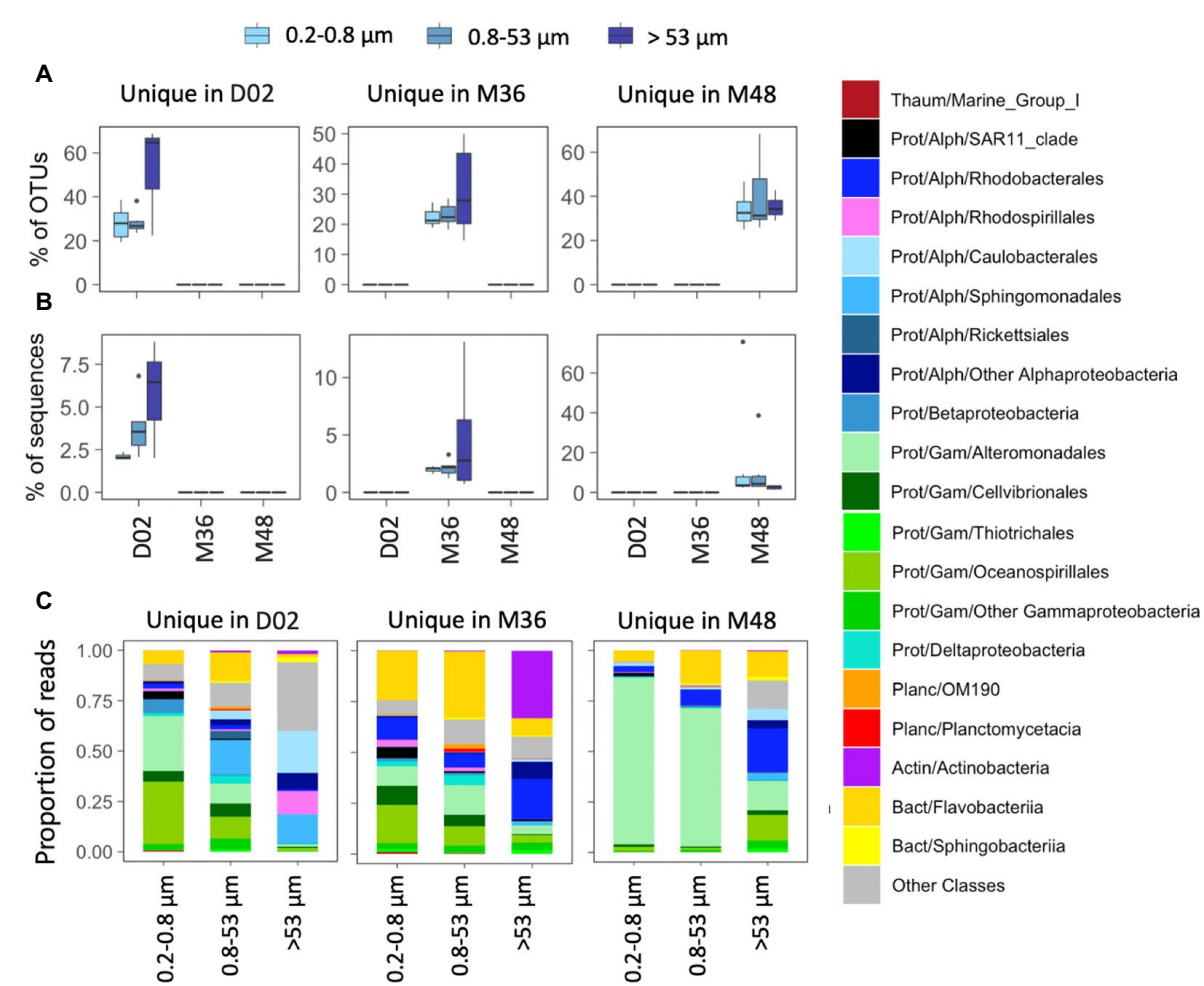
**(A,B)** Contribution in terms of % OTUs **(A)** or sequences **(B)** of those OTUs detected exclusively in one station (“unique” OTUs). **(C)** Taxonomic composition of unique OTUs in the three size-fractions. The classification was performed at the class level although in some cases the main orders are also indicated. The corresponding phyla and classes are indicated in each case: Thaum, thaumarchaeota; Actin, actinobacteria; Bact, bacteroidetes; Planc, planctomycetes; Prot, proteobacteria; Alph-, *Alphaproteobacteria* and Gam-, *Gammaproteobacteria*. Note the different scales of the Y axes.

All studied communities harbored a relatively large fraction of OTUs that were exclusive from a given sampling station (i.e., unique OTUs, range 14–69% of community OTUs, Figure 4A), although these accounted for a smaller fraction of local community sequences (range 1–13%) except for two communities from M48 where unique OTUs comprised 39 and 76% of local sequences (Figure 4B). In stations D02 and M36, the highest percentage of unique OTUs was found in some of the communities associated with the largest particles, whereas in M48, a similar contribution of unique OTUs was found across size-fractions (Figure 4A). These unique OTUs belonged to different orders within *Gammaproteobacteria* (mostly *Alteromonadales*, *Cellvibrionales*, and *Oceanospirillales*), *Alphaproteobacteria* (*Sphingomonadales* and *Rhodobacterales*) and *Flavobacteriia* across most size-fractions, except for a large contribution of Actinobacteria unique OTUs in the large particles from station M36 (Figure 4C).

**Figure 5:**
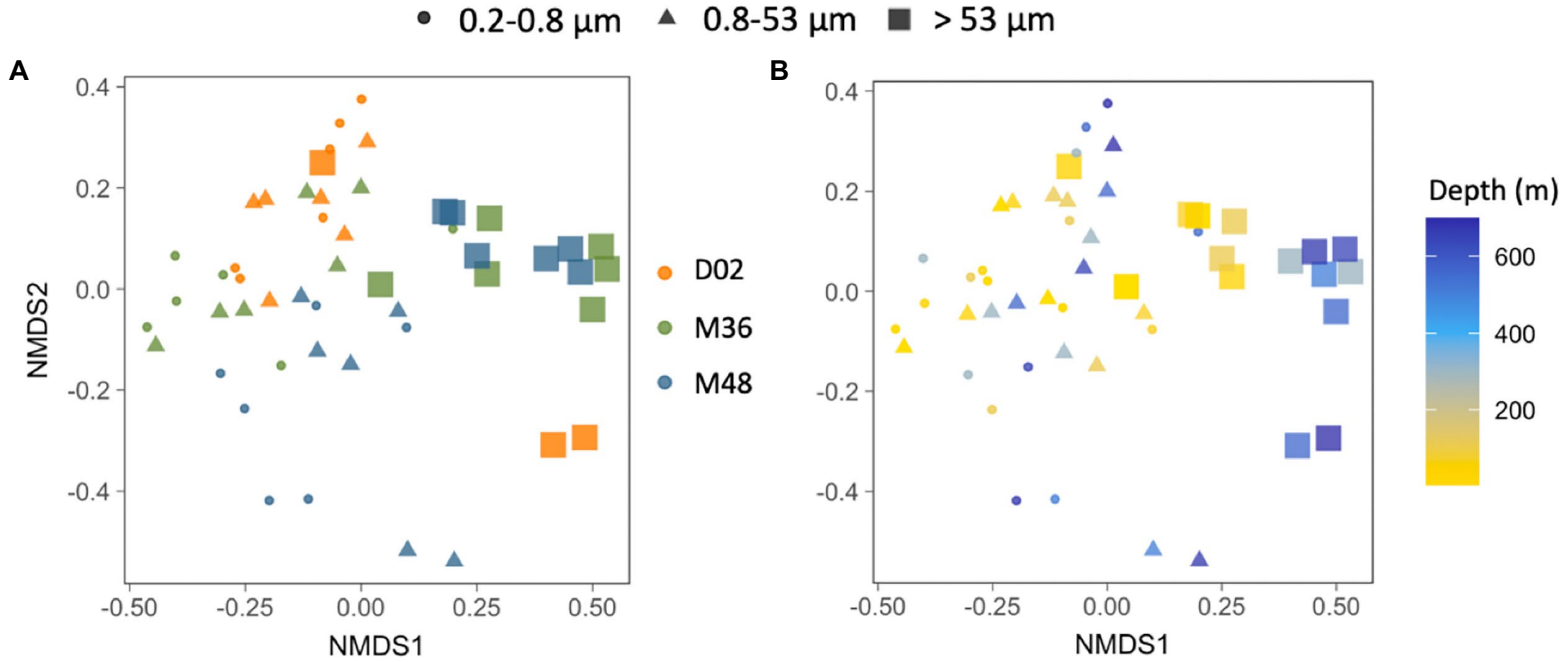
**(A,B)** Non-multidimensional scaling analysis (NMDS) based on the Bray–Curtis dissimilarity between all the studied prokaryotic communities, color-coded by sampling station **(A)** or by sampling depth **(B)** and indicating the different size-fractions. Stress value = 0.18.

A non-metric multidimensional scaling (NMDS) analysis showed that, when pooling all samples together, communities from the largest particles differed from assemblages associated with the other two size-fractions, which were more similar to each other. These differences between size-fractions (Figures 5A,B, ANOSIM_bysize_ *R* = 0.46, *p* < 0.001) were more important than the spatial differences among stations (Figure 5A, ANOSIM_bystation_ *R* = 0.14, *p* < 0.001) and the vertical differences throughout the water column (Figure 5B, ANOSIM_bydepth_ *R* = 0.18, *p* < 0.005).

**Figure 6:**
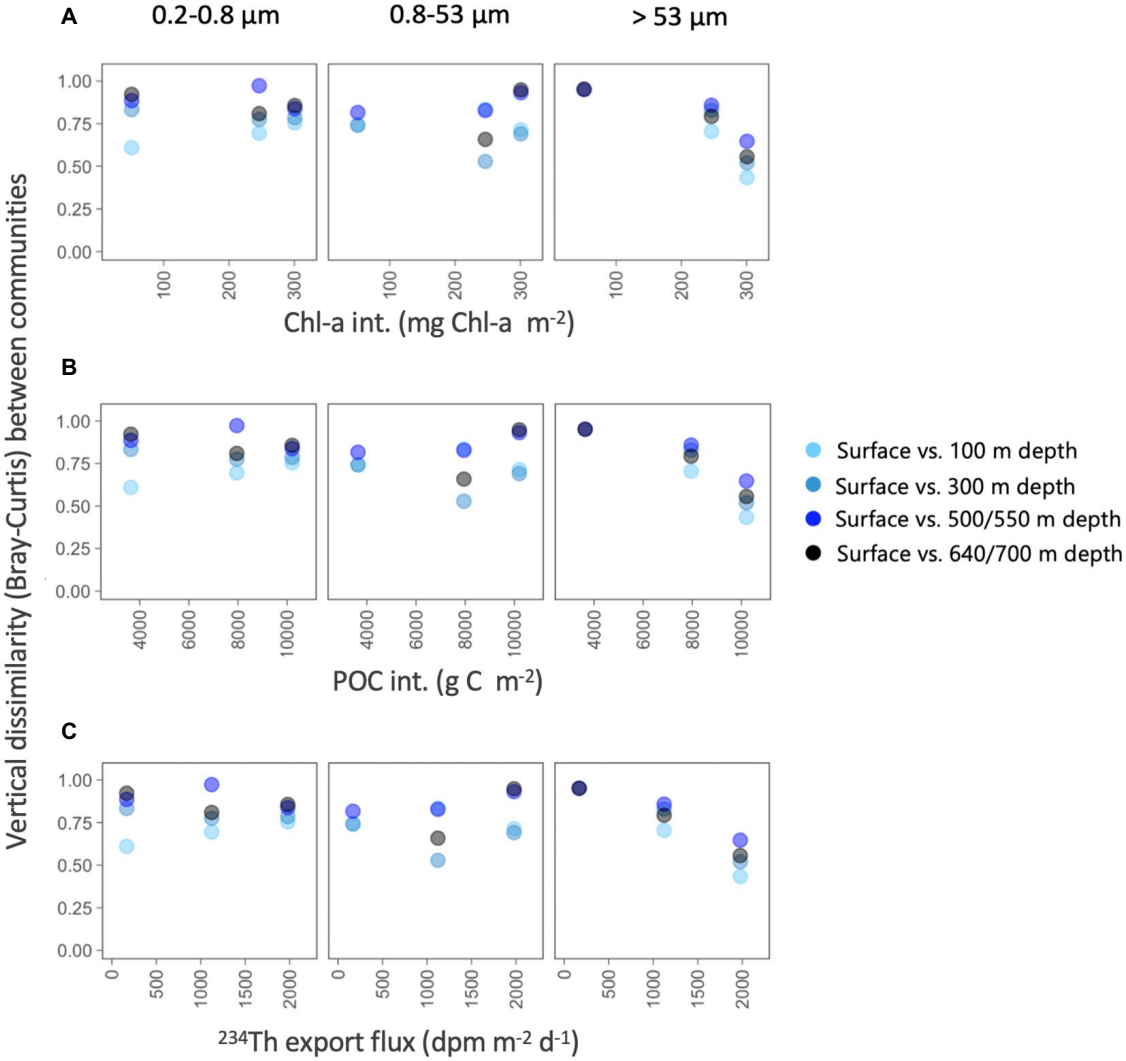
Variation in vertical taxonomic differences (Bray–Curtis dissimilarity) between surface and each of the deeper prokaryotic communities (100–700 m depth), along gradients in depth-integrated chlorophyll-a concentration, depth-integrated POC concentration and estimated ^234^Th export fluxes for each of the three size-fractions.

To determine whether the particle flux explained the vertical differences within each size-fraction, we estimated the Bray–Curtis dissimilarity between the surface (5 m) and each of the deeper prokaryotic communities and explored how these vertical differences varied along environmental gradients related to surface productivity (using Chl-a and POC as proxies) and particle flux (Figure 6). For the two smaller size-fractions, we found that, as expected, the vertical dissimilarity with the surface communities increased with depth, but no clear patterns were found along the productivity gradients (Figure 6). However, we found that the vertical differences between surface and all deeper prokaryotic communities associated with the largest particles decreased along a gradient of Chl-a and POC stocks, and with increasing ^234^Th export fluxes, and this happened between all depths, suggesting that communities from the large size-fraction were more similar along the water column in situations of higher surface productivity and ^234^Th export fluxes.

**Figure 7:**
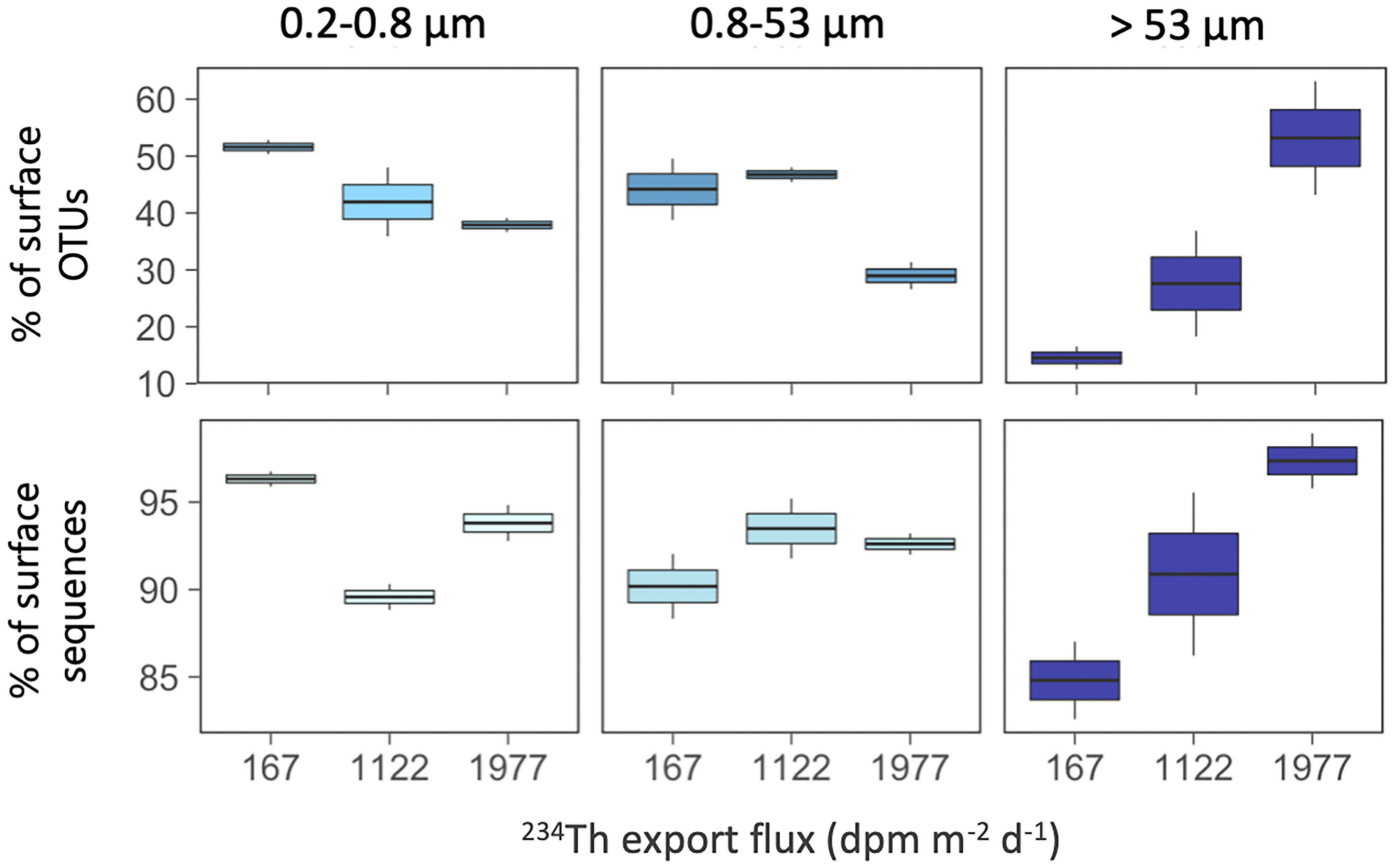
Contribution of surface-derived OTUs (top panels) and their sequences (bottom panels) in mesopelagic communities (≥500 m) along the gradient of ^234^Th export fluxes. Surface-derived OTUs are those mesopelagic OTUs that were also present in surface waters (≤100 m). Note the different scales of the Y axes.

Finally, we explored whether the contribution of surface-derived OTUs (i.e., OTUs with presence in any of the surface waters studied, i.e., ≤100 m) to the communities of the mesopelagic samples changed along these surface gradients related to productivity and particle fluxes (Figure 7). We found that mesopelagic communities in the >53 μm particles had a larger proportion of surface-derived OTUs along the increasing ^234^Th flux gradient, ranging from 13 to 63% of the mesopelagic OTUs in this size-fraction (Figure 7A), accounting for 83 to 99% of the local sequences (Figure 7B). In other words, the higher the particle export flux, the higher the proportion of surface-derived taxa present in the large mesopelagic particles. This tendency was not observed for the other two size-fractions, although in general, we found that all mesopelagic communities harbored a large fraction of OTUs (range 28–52%) and of sequences (range 88–97%) which had first been detected in the overlying surface waters (Figure 7).

## 4. Discussion

The seasonally ice-covered coastal regions of Antarctica host a disproportionately large fraction of its primary production relative to their surface area and support complex food webs with a diversity of upper trophic levels (Arrigo and van Dijken, 2003; Karnovsky et al., 2007), yet these marginal seas and polynyas are heavily understudied. Modeling efforts suggest that about 30% of the Southern Ocean primary production is exported below the euphotic zone (Henson et al., 2012), but high variability has been reported at a smaller spatial scale. For example, in the Amundsen Sea Polynya, the reported most productive coastal Antarctic polynya, bacterial respiration remineralized >95% of the surface-derived particulate organic carbon within the upper 400 m, leading to very low export efficiencies and minimal carbon sequestration (Ducklow et al., 2015; Lee et al., 2017). Particles sinking from the surface ocean undergo remineralization by microbial communities colonizing them, leading to an attenuation of the flux of organic matter toward the deep ocean (Buesseler and Boyd, 2009). This suggests that different particle-attached microbial communities may sway the efficiency of the biological carbon pump in these productive systems, but very few studies have characterized prokaryotic communities associated with particles from Antarctic polynyas. Here, by coupling particle export fluxes with vertical variations in prokaryotic communities associated with different particle size-fractions, we show a large influence of surface productivity on the structure of mesopelagic particle-attached communities, suggesting that expected changes in Antarctic productivity due to global change may have large impacts on carbon sequestration and deep-sea microbiota.

Polynyas, despite sharing similarities in size or location, can be biogeochemically very different. These differences result from a myriad of physicochemical processes related to, among others, mixing, ice melting, ocean currents, interaction with the continental shelf, etc., and can lead to local differences in the timing, magnitude, and extension of phytoplankton blooms in these ecosystems even in closely located sites. Actually, despite their relative spatial proximity, the two polynyas sampled for this study were markedly different in terms of physicochemical and biological characteristics. As discussed in Moreau et al. (2019), the lower phytoplankton biomass and net community production in the Dalton Polynya was a consequence of the different water masses present in both polynyas, with warm modified Circumpolar Deep Water (rich in iron, an essential micronutrient that limits the growth of phytoplankton in the Southern Ocean) being widespread down to the ocean floor at Dalton, whereas it was found at shallower depths in the Mertz. Nutrients measured in both polynyas also indicated differences in the phytoplanktonic community between them, as the depletion of silica in surface waters of the Mertz Polynya coincided with the dominance of the diatoms *Fragilariopsis curta* and *F. cylindrus*, whereas small flagellates (mainly *Phaeocystis antarctica*) dominated the community in the Dalton Polynya (Moreau et al., 2019). Within the Mertz Polynya, station M36 presented clearly warmer and saltier surface waters compared to M48 and had a slightly smaller fluorescence peak that was concentrated in the upper 20 m vs. a fluorescence peak that extended down to 100 m at station M48 (Supplementary Figure S1B). M36 also had lower Chl-a and POC concentrations and stocks as well as lower ^234^Th export flux, thus creating a gradient of surface “productivity” and particle export across the three stations (D02 < M36 < M48).

### 4.1. Prokaryotic communities in the Dalton and Mertz polynyas

We characterized the prokaryotic assemblages associated with three size-fractions from surface to mesopelagic waters in three stations to explore whether the different surface conditions explained variations in the prokaryotic communities. The largest size-fractions harbored the prokaryotic communities with the lowest number of OTUs at the three stations. This pattern is contrary to that found in previous size-fractionation studies in the Mediterranean where prokaryotic richness was shown to increase with particle size and was attributed to higher niche availability (Mestre et al., 2017, 2020) but agrees with the decrease in richness from free-living to particle-attached found during the global Malaspina ocean expedition (Salazar et al., 2015; Mestre et al., 2018).

The communities from the three size-fractions showed pronounced vertical variations in OTU number, but these did not seem related to variations in surface productivity. For example, whereas at stations D02 (the less productive) and M48 (the most productive), richness decreased from the subsurface to mesopelagic waters, at station M36, smaller vertical changes were observed, except for a subsurface (~20–50 m) peak in richness which was observed across the three stations. This contrasts with studies in the Amundsen Sea polynya showing depth-driven increases in richness in free-living prokaryotic communities (Kim et al., 2014; Richert et al., 2019), and highlights a complexity in the nature of the particles and the associated microbial communities depending on the location and likely the surface conditions.

We found a dominance of groups like *Alphaproteobacteria*, *Gammaproteobacteria*, and *Flavobacteriia* which are the most common ones in the polar oceans (Ghiglione et al., 2012), although their abundances varied depending on the size-fraction and depth. Within them, groups like *Colwellia*, *Pseudoalteromonas*, *Alcanivorax* (*Gammaproteobacteria*), *Polaribacter* (*Flavobacteriia*), and *Pseudophaeobacter* (*Alphaproteobacteria*) were the most abundant. Previous reports in the Amundsen Sea polynya have shown a surface dominance of fast-growing copiotrophs including members of *Flavobacteriia*, *Polaribacter*, *Gammaproteobacteria* SAR92, and Oceanospirillaceae and/or of different members within *Flavobacteriia* and Alpha- and *Gammaproteobacteria* in mesopelagic waters (Delmont et al., 2014; Kim et al., 2014; Richert et al., 2015, Choi et al., 2016). Marine Flavobacteria have been described as major components of marine aggregates (e.g., Zhang et al., 2007), with abundances of particle-associated Flavobacteria suggested to be related to enhanced primary production (Abell and Bowman, 2005). However, none of these studies conducted particle size-fractionation analyses, so very little is known about the particle-attached microbiome of Antarctic polynyas. In our study site, we found that their abundance drastically decreased in the largest size-fraction, yet there was a higher abundance of *Flavobacteriia* in the Mertz stations (more productive) compared to the Dalton station. We found a remarkable dominance of *Alphaproteobacteria* in the largest size-fractions across most our samples, which comprised mostly the genera *Brevundimonas* and *Sphingorhabdus* at the Dalton station, and *Pseudophaeobacter* in the case of the two Mertz stations, pointing to large differences in the particle-associated communities between both polynyas. Although *Alphaproteobacteria* are usually found as free-living (Salazar et al., 2016; Mestre et al., 2020), they have been shown to dominate large particles in some areas of the ocean (Mestre et al., 2018) and genera such as *Brevundimonas* have been detected in late stages of particle colonization (Pelve et al., 2017). The differences in the community composition of the large particles from deep waters between the two polynyas could be due to differences in the origin and composition of particles, as well as to differences in the prokaryotic surface inocula (Mestre et al., 2018; Ruiz-González et al., 2020). Actually, we found that all studied communities harbored a relatively high fraction of OTUs that were exclusive from each station (unique OTUs), and this proportion was higher in some of the largest size-fractions from the Dalton D02 and the Mertz M36 stations. This supports that the local physicochemical or biotic conditions established within each polynya may select for specific taxa or that there is dispersal limitation of species across the sampled sites. However, in general, these unique OTUs represented a small fraction of communities in most cases and communities were dominated by taxa that showed presence in both polynyas.

Surface water properties are known to mold marine microbial communities in Antarctic waters (Piquet et al., 2011; Ghiglione et al., 2012; del Negro et al., 2018). For example, Wilkins et al. (2013) conducted a metagenomic survey from Hobart to the Mertz Glacier and found different taxonomic and functional microbial assemblages north and south of the Polar Front. The differences observed concurred with the more oligotrophic characteristics found north of the Polar Front compared to the usually enhanced primary production observed in summer in the Antarctic coastal areas. However, and despite the relevance of Antarctic polynyas in carbon cycling, few studies have characterized their pelagic prokaryotic communities, and most have focused on polynyas from the Amundsen Sea and on the free-living fraction of prokaryotic communities (e.g., Delmont et al., 2014; Kim et al., 2014; Richert et al., 2015, 2019; Choi et al., 2016). These studies have reported vertical differences in the abundances of several prokaryotic groups at different areas within the polynya (Kim et al., 2014) as well as clear linkages between phytoplankton communities and prokaryotic assemblages (Delmont et al., 2014; Kim et al., 2014; Richert et al., 2019). For example, Kim et al. (2014) found that free-living bacterioplankton abundance was strongly correlated with the abundance of *Phaeocystis* spp. and diatoms, and Richert et al. (2019) reported increases in surface bacterioplankton abundance as chlorophyll increased in surface waters during a phytoplankton bloom. Delmont et al. (2014) found that groups such as the SAR92 clade and *Colwellia*, which dominated different size-fractions and depths in our study, were prevalent particle-attached prokaryotic communities at the surface and at 250 m depth, respectively, in the Amundsen Polynya during a *Phaeocystis* bloom, suggesting that they may play important roles at different stages of the bloom. Our results also show highly different communities between surface and deep microbial communities from the two Antarctic polynyas, but the magnitude of these vertical changes differed largely between the studied sites and size-fractions.

### 4.2. Links between surface and mesopelagic communities

The differences in surface conditions and phytoplankton communities (*Phaeocystis* vs. diatoms) between the two studied polynyas translated into different particle fluxes (estimated through ^234^Th export fluxes) and carbon export efficiency, which were significantly lower in the Dalton Polynya compared with the Mertz Polynya (5% vs. 15%, respectively; see Ratnarajah et al., 2022). Estimates of particle export using ^234^Th proxy have not been previously obtained in Antarctic polynyas, but based on basin-wide and global compilations (e.g., Le Moigne et al., 2013; Owens et al., 2015; Puigcorbé et al., 2017b) the ^234^Th export flux estimated in D02 is very low, characteristic of oligotrophic and low productive ocean areas, whereas the flux at M36 is within the range of values found in temperate areas and the flux observed at M48 resembles fluxes observed under bloom conditions. POC export efficiencies (i.e., fraction of net primary production, NPP, that is exported to certain depth: POC flux/NPP*100) were found to be 3 times lower in the Dalton Polynya (5%) compared to the Mertz Polynya (15%), where transfer efficiencies down to 300 m were >80% (Ratnarajah et al., 2022).

The structure of phytoplankton communities impacts the particles produced in surface waters, consequently affecting carbon export rates (Siegel et al., 2014 and references therein). These sinking particles are known to influence the ecology and assembly of deep-sea microbial communities, not only by delivering surface-derived organic carbon (Arístegui et al., 2009; Herndl and Reinthaler, 2013), but also by directly transporting surface prokaryotes, some of which may colonize deeper waters (Mestre et al., 2018; Wenley et al., 2021). Despite the gradient in surface conditions and particle export, no clear clustering of the deep prokaryotic communities was observed by station (Figure 5), suggesting that the environmental characteristics were not different enough to lead to strong taxonomic variation between the three stations, not even between the highly contrasting Dalton and Mertz polynyas, when all samples were considered together. However, we found that the vertical dissimilarity between surface and deep communities was reduced (i.e., similarity was higher) when there was an increase in the Chl-a and POC inventories or when the flux of particles increased; in other words, the higher the production and export of particles (using Chl-a, POC and ^234^Th deficit as proxies), the more taxonomically similar were the surface and mesopelagic prokaryotic communities associated with the largest particles. It is worth mentioning that these correlations were observed for the >53 μm size-fraction only. This agrees with the observation that particles of large sizes are more efficient vectors of diversity from the surface to the deep ocean (Mestre et al., 2018), and also with the increases in vertical similarity between surface and meso- or bathypelagic particle-attached communities with increasing surface productivity observed across the global ocean (Ruiz-González et al., 2020).

The estimated variations in particle export fluxes also coincided with a higher contribution of surface-derived OTUs to mesopelagic communities in the largest size-fractions, with the station with the highest particle export fluxes having higher contributions of surface OTUs, supporting the hypothesis of a direct transport of surface bacteria down to mesopelagic waters in highly productive Antarctic polynyas. Similarly, a recent study by Valencia et al. (2022) conducted in the Eastern North Pacific, showed that the taxa found in sinking particles were more similar to the taxa found in the upper water column in highly productive areas than in oligotrophic waters. Also, a global expedition showed that the deep-sea prokaryotic taxa with presence in the overlying surface waters were found to be mainly typical copiotrophs or eukaryote-associated groups (Ruiz-González et al., 2020). All these support that particles reaching deeper waters may comprise larger, fast-sinking material of recent phytoplankton origin that may avoid remineralization processes occurring in shallow layers (Agusti et al., 2015; Grabowski et al., 2019), representing a direct inoculation of surface particle-attached prokaryotic taxa into deep waters, and explaining the increase in the contribution of surface OTUs to large mesopelagic particles in station M48, the one with the highest estimated particle export flux.

Several previous studies have also evidenced tight linkages between the surface and deep-ocean microbial communities. For example, changes in bathypelagic prokaryotic abundance or activity have been related to high carbon fluxes or surface primary production in different oceanic sites (Nagata et al., 2000; Hansell and Ducklow, 2003; Tamburini and Garcin, 2003; Yokokawa et al., 2013) and taxonomic shifts in deep-sea communities have been linked to spatial or temporal variations in surface conditions related to particle formation and sinking (Cram et al., 2015; Parada and Fuhrman, 2017; Santoro et al., 2017; Ruiz-González et al., 2020; Wenley et al., 2021). To our knowledge, however, very few studies have compared the vertical changes in prokaryotic community composition with particle export fluxes, except for the study by Poff et al. (2021), who found that in situations of elevated carbon flux events in the North Pacific Subtropical Gyre, particle-attached bacteria reaching abyssal depths had surface water origins, and Fadeev et al. (2021), who observed that ice-covered areas in the Arctic had higher carbon export and were also associated with lower dissimilarity between surface and deep sea microbial clades. Our study is in line with these recent findings and provides the first direct attempt to link simultaneous analyses of particle flux with the microbiome of particles in Antarctic polynyas. Our results suggest that changes in surface phytoplankton assemblages and/or productivity may strongly affect the deep-ocean microbial communities associated with the largest sinking particles.

## 5. Conclusion

The opening timings and size of Antarctic polynyas are likely to increase in coming years due to climate change, which will lead to changes in phytoplankton blooms (Montes-Hugo and Yuan, 2012; Deppeler and Davidson, 2017), as well as changes in the structure and function of microbial communities in these highly productive ecosystems (Piquet et al., 2011; Hörstmann et al., 2022) with yet unknown consequences for ecosystem functioning and carbon export production (Fan et al., 2020). Here, we evidenced that the vertical structuring of particle-attached microbial communities from two contrasting polynyas differed markedly depending on surface conditions. In general terms, there was a segregation of communities between surface and mesopelagic waters, yet differences were less strong at certain stations (M48) and particularly for communities associated with the largest size-fraction.

*Alphaproteobacteria*, *Gammaproteobacteria*, and *Flavobacteriia* dominated all communities, but different genera were found at different depths and size-fractions. The observed vertical, spatial, and size-fraction dependent variations in prokaryotic community composition support that there are different microbial niches within and across the studied polynyas. Estimates of particle export fluxes based on ^234^Th coincided with higher chlorophyll-a and particulate organic carbon concentrations and stocks. We found that when the particle flux was higher, mesopelagic prokaryotic communities from the largest size-fraction were more similar to those found in sunlit waters and contained higher proportions of surface-derived taxa, evidencing an intense downward transport of surface bacteria mediated by the largest particles. In consequence, surface conditions will influence differently the deep ocean prokaryotic assemblages depending on the size, origin, and composition of the sinking material. To our knowledge, this is the first direct evidence of compositional differences in particle-attached assemblages linked to the magnitude of the particle flux in Antarctic polynyas. Further research is needed to stablish a mechanistical model that could allow to predict bacterial community structures based on particle export and surface productivity.

## Data availability statement

The raw sequence data have been deposited in the Figshare data repository, together with the non-rarefied OTU table, the taxonomy table and the environmental data used in this study, doi: 10.6084/m9.figshare.21385215.v1.

## Author contributions

VP, JG, and PM participated in the design of the sampling scheme. VP performed the sampling and sample processing. VP and CR-G compiled the needed data, analyzed the data, and wrote this article. All authors contributed to the article and approved the submitted version.

## Funding

The project that gave rise to these results received the support of a fellowship to VP from “la Caixa” Foundation (ID 100010434) and from the European Union’s Horizon 2020 research and innovation program under the Marie Skłodowska-Curie grant agreement no 847648 (fellowship code LCF/BQ/PI21/11830020). VP also received funding from Edith Cowan University (G1003456) and from the School of Science at Edith Cowan University (G1003362) to support this work. CR-G was supported by the grants RTI2018-101025-B-I00 and a Ramon y Cajal contract (RYC2019-026758-I) and JG by grants CTM2015-70340-R and PID2021-125469NB-C31 of the Spanish Ministry of Science and Innovation and by the Generalitat de Catalunya Consolidated Research Group 2017SGR/1568.

## Conflict of interest

The authors declare that the research was conducted in the absence of any commercial or financial relationships that could be construed as a potential conflict of interest.

## Publisher’s note

All claims expressed in this article are solely those of the authors and do not necessarily represent those of their affiliated organizations, or those of the publisher, the editors and the reviewers. Any product that may be evaluated in this article, or claim that may be made by its manufacturer, is not guaranteed or endorsed by the publisher.
