# Supplementary material for: Impact of particle flux on the vertical distribution and diversity of size-fractionated prokaryotic communities in two East Antarctic polynyas
Source: Front Microbiol. 2023 Feb 23;14:1078469. doi: 10.3389/fmicb.2023.1078469 (PMC9995690; doi:10.3389/fmicb.2023.1078469)
Supplement: Supplementary file 1 [file Data_Sheet_1.pdf]

Suppl. Fig. 1: A) Map of the location of the polynyas and the sampled station. B) Profiles of temperature, salinity, fluorescence, and inorganic nutrients' concentrations at the three study sites.

A)

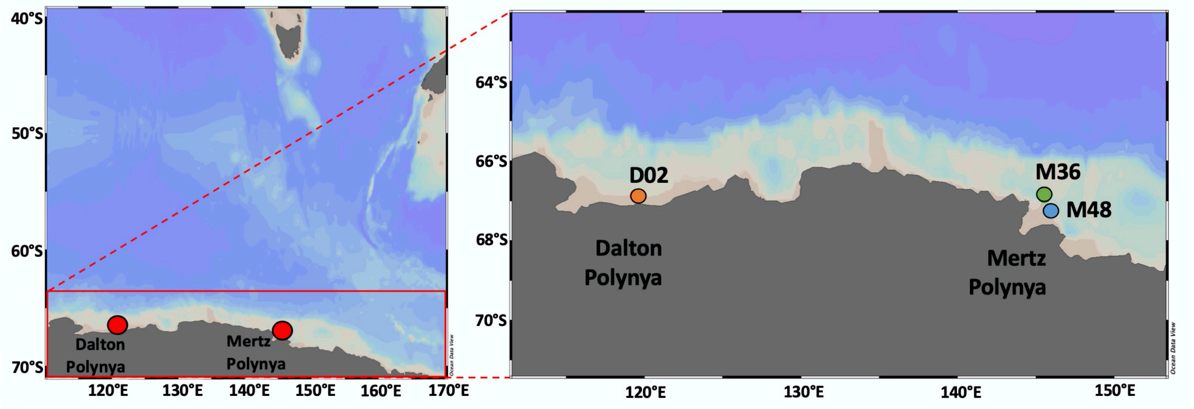

B)

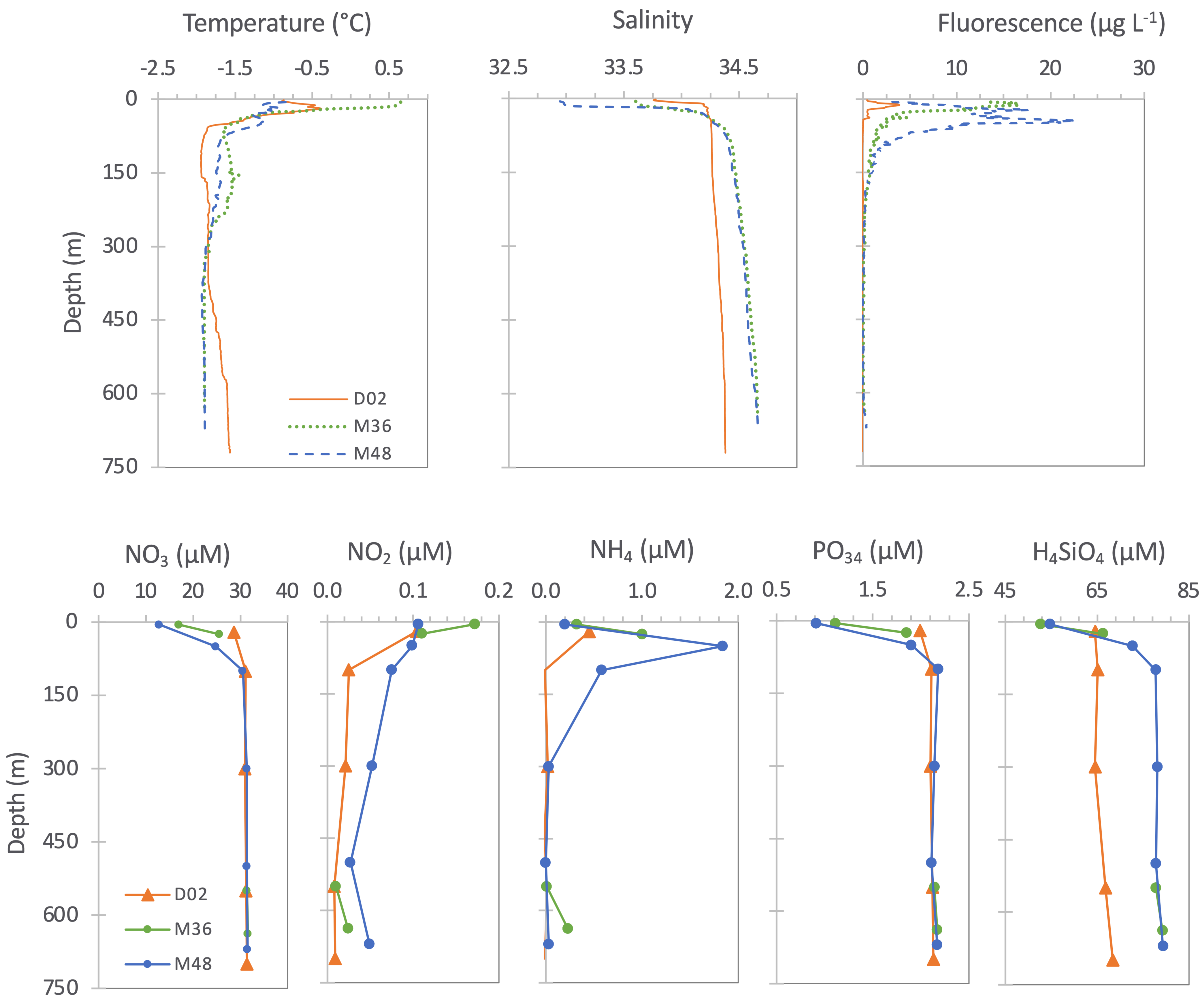

Suppl. Figure 2. Contribution of the main bacterial genera (>1% of the sequences in the dataset) across stations, depths and size fractions. The corresponding phyla and classes are indicated in each case: Thaum, Thaumarchaeota; Actin, Actinobacteria; Bact, Bacteroidetes; Planc, Planctomycetes; Prot, Proteobacteria. Alph-, Gam-, Alphaproteobacteria and Gammaproteobacteria; Flav, Flavobacteriia; MGI, Marine Group I.

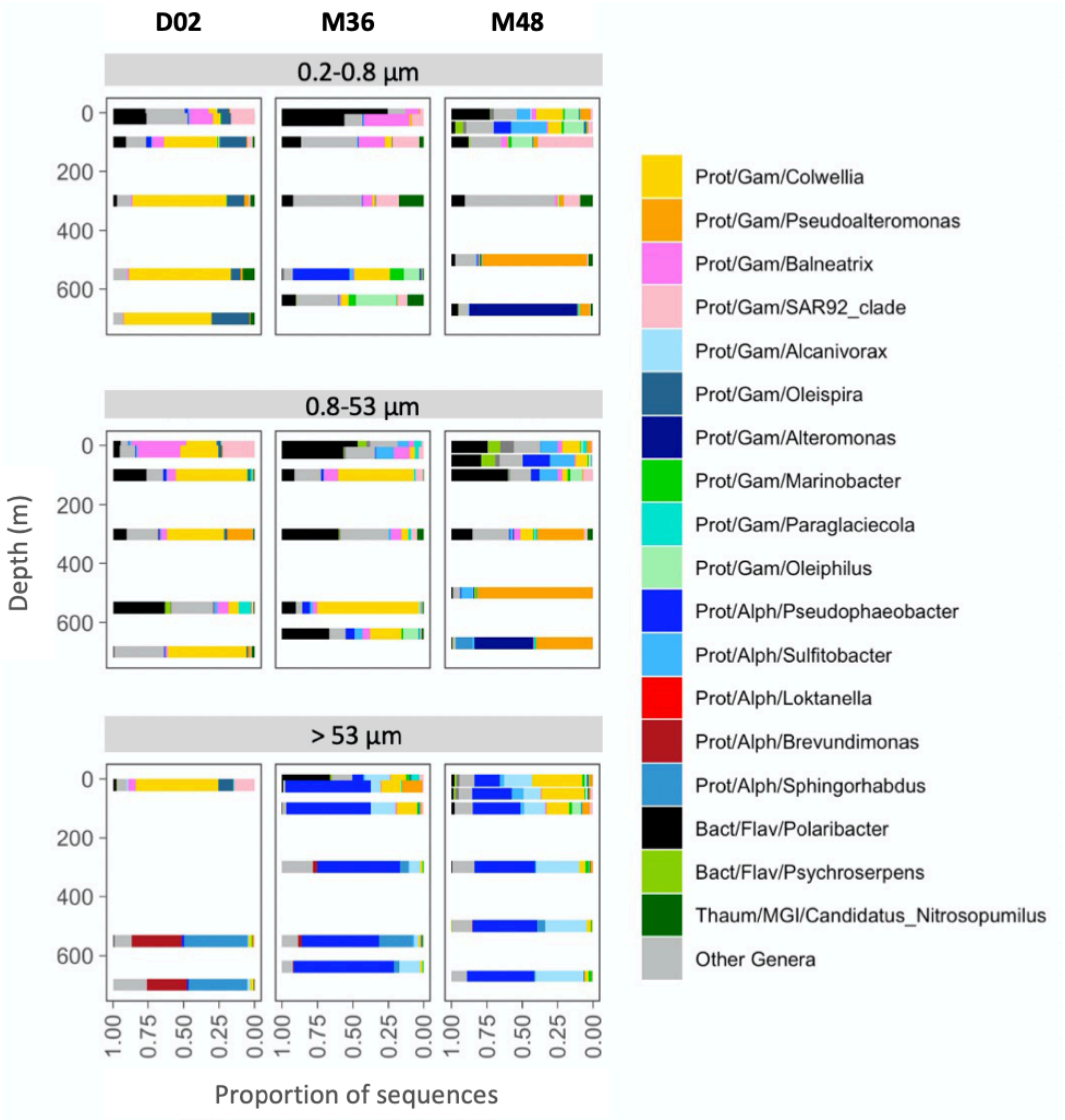

This is a provisional file, not the final typeset article
